# The Metabolic Bone Disease X-linked Hypophosphatemia: Case Presentation, Pathophysiology and Pharmacology

**DOI:** 10.3390/life11060563

**Published:** 2021-06-15

**Authors:** Jon Vincze, Brian W. Skinner, Katherine A. Tucker, Kory A. Conaway, Jonathan W. Lowery, Julia M. Hum

**Affiliations:** 1 College of Osteopathic Medicine Division of Biomedical Science, Marian University, 3200 Cold Spring Rd., Indianapolis, IN 46222, USA; jvincze114@marian.edu (J.V.); Kconaway713@marian.edu (K.A.C.); 2 Bone & Muscle Research Group, Marian University, 3200 Cold Spring Rd., Indianapolis, IN 46222, USA; 3 College of Osteopathic Medicine Division of Clinical Sciences Marian University, 3200 Cold Spring Rd., Indianapolis, IN 46222, USA; bskinner@marian.edu; 4 Leighton School of Nursing, Marian University, 3200 Cold Spring Rd., Indianapolis, IN 46222, USA; KATucker@marian.edu

**Keywords:** metabolic bone disease, burosumab, KRN23, X-linked hypophosphatemia, Crysvita^®^

## Abstract

The authors present a stereotypical case presentation of X-linked hypophosphatemia (XLH) and provide a review of the pathophysiology and related pharmacology of this condition, primarily focusing on the FDA-approved medication burosumab. XLH is a renal phosphate wasting disorder caused by loss of function mutations in the *PHEX* gene (phosphate-regulating gene with homologies to endopeptidases on the X chromosome). Typical biochemical findings include elevated serum levels of bioactive/intact fibroblast growth factor 23 (FGF23) which lead to (i) low serum phosphate levels, (ii) increased fractional excretion of phosphate, and (iii) inappropriately low or normal 1,25-dihydroxyvitamin D (1,25-vitD). XLH is the most common form of heritable rickets and short stature in patients with XLH is due to chronic hypophosphatemia. Additionally, patients with XLH experience joint pain and osteoarthritis from skeletal deformities, fractures, enthesopathy, spinal stenosis, and hearing loss. Historically, treatment for XLH was limited to oral phosphate supplementation, active vitamin D supplementation, and surgical intervention for cases of severe bowed legs. In 2018, the United States Food and Drug Administration (FDA) approved burosumab for the treatment of XLH and this medication has demonstrated substantial benefit compared with conventional therapy. Burosumab binds circulating intact FGF23 and blocks its biological effects in target tissues, resulting in increased serum inorganic phosphate (Pi) concentrations and increased conversion of inactive vitamin D to active 1,25-vitD.

## 1. Case Presentation

A twenty-month-old male presented to his pediatrician’s office with a chief complaint of delayed walking milestone. The child was born vaginally following an uncomplicated pregnancy and breastfed until twelve months of age when he was transitioned to whole cow’s milk and continued solids. Postnatal screening was unremarkable. The child was twentieth percentile for length, thirtieth percentile for weight, fortieth percentile for occipital-frontal circumference, appeared pleasant while seated, and vitals were within normal ranges. Physical exam revealed bilateral valgus knees and a pain response while standing. The abdomen was soft, round, free of masses and the child soiled four to five diapers per day and had a daily bowel movement. Family history was unremarkable. Laboratory studies indicated elevated serum levels of FGF23, hypophosphatemia, normocalcemia, and low serum levels of 1,25-dihydroxyvitamin D (1,25-vitD) and hyperphosphaturia was noted via urinalysis. Genetic testing revealed a loss-of-function mutation in *PHEX* (phosphate-regulating gene with homologies to endopeptidases on the X chromosome) which led to a diagnosis of the metabolic bone disease X-linked hypophosphatemia (XLH).

## 2. X-linked Hypophosphatemia

XLH is the most common form of heritable rickets and its diagnosis is generally based on physical exam, biochemical analyses of serum and/or plasma, imaging tests, and family history. XLH is a renal phosphate wasting disorder caused by loss of function mutations in the *PHEX* gene [1,2,3]. *PHEX* is a member of the M13 family of membrane-bound metalloproteases, and its expression is highest in bone cells, specifically osteoblasts and osteocytes [4]. Mutation in the *PHEX* gene product leads to increased serum levels of FGF23. FGF23 is a potent phosphaturic agent, and tubular phosphate reabsorption is disrupted in patients with XLH via downregulation of sodium-phosphate cotransporters in the proximal renal tubule [1]. Other biochemical findings typically include elevated low serum phosphate levels, increased fractional excretion of phosphate, normocalcemia and inappropriately low or normal 1,25-vitD [5]. Serum levels of parathyroid hormone (PTH) vary. Short stature in patients with XLH is due to chronic hypophosphatemia and, as adults, patients with XLH often experience joint pain and osteoarthritis from childhood skeletal deformities, fractures, enthesopathy, spinal stenosis, and hearing loss [6,7,8].

XLH is a debilitating lifelong burden that is due to elevated bioactive FGF23. FGF23 was initially identified through gain-of-function mutations in a different form of rickets, the rare heritable disorder autosomal dominant hypophosphatemia rickets (ADHR) [9]. Prior to this discovery, phosphate homeostasis was thought to be maintained through PTH and 1,25-vitD. Under normal physiological conditions, elevations in serum phosphate or active 1,25-vitD stimulate upregulation of FGF23 and promote the release of the full-length intact FGF23 protein that targets downstream tissues. FGF23 acts directly on the kidneys to promoting phosphate excretion and indirectly through its effects on 1,25-vitD. Additionally, FGF23 suppresses the transcription of the genes encoding sodium-phosphate cotransporters [1].

## 3. Pharmacological Management of XLH

Historically, treatment for XLH was limited to oral phosphate supplementation, 1,25-vitD supplementation, and surgical intervention for cases of severely bowed legs [10]. However, in April 2018, the United States FDA approved burosumab (Crysvita^®^, Ultragenyx, Novato, CA, USA), which is a human monoclonal IgG1 antibody against FGF23, for treatment of adults and children with XLH. Burosumab was granted both breakthrough therapy designation and orphan drug designation by the FDA [11]. Burosumab binds circulating intact FGF23 and blocks its biologic effects in target tissues, resulting in increased serum inorganic phosphate (Pi) concentrations and increased conversion of inactive vitamin D to active 1,25-vitD [11].

The efficacy of burosumab in both pediatric and adult patients with hypophosphatemia have been compared to placebo and conventional care in clinical trials, the results of which are summarized in Table 1. All adult and pediatric trials found burosumab significantly increased Pi to within normal levels and sustain that increase across the length of the study with 94.1% of adults achieving a Pi above the lower limit of normal (LLN) in one study (compared with 7.6% of those receiving placebo, *p* < 0.001) [12]. Furthermore, pediatric patients experienced a mean increase in Pi ranging from 0.84–0.92 mg/dL across various trials [10,13,14]. Many clinical trials examined secondary markers of bone health—including 1,25-vitD levels, radiographic assessments using the Thacher’s radiographic severity score (TRSS) along with the Radiographic Global Impression of Change (RGI-C) and found significant improvements.

A histomorphometric study including 14 adult subjects with XLH found significant improvement in bone formation and reduced bone resorption with burosumab leading to lower osteoid thickness (−32%), osteoid surface/bone surface (−26%), and mineralization lag time (−83%) after 48 weeks of therapy [15]. Additionally, burosumab significantly improved several parameters on the Brief Pain Inventory (BPI) including mean pain severity score (5.1 at baseline vs. 3.4, *p* = 0.0013), mean worst pain score (6.6 vs. 4.9, *p* = 0.0054), and mean pain interference score (5.2 vs. 4.0, *p* = 0.0060) along with a reduction in the BPI mean fatigue score (6.8 vs. 5.5, *p* = 0.0156). This study also identified four patients with active pseudofractures at the beginning of the study. At week 12, two demonstrated complete healing and the other two healed partially. At the conclusion of the study, 75% had healed completely, but the other pseudofractures could not be assessed due to lack of radiographic imaging [15].

A larger double-blind, placebo-controlled trial of 134 adults also examined objective markers related to the quality of life of subjects with XLH along with fracture healing as secondary markers of efficacy. Utilizing the Western Ontario and McMaster Universities Arthritis (WOMAC) index, an 8% reduction of stiffness reached statistical significance when compared to placebo (mean difference of −8.1, *p* = 0.012), though the WOMAC physical function subscale and the BPI worst pain indices failed to achieve significance after Hochberg multiplicity adjustments. However, both these measurements reached significance in Ruppe et al., albeit with a smaller sample size [12,16]. Notably, 43.1% of active fractures were fully-healed in the burosumab group compared with only 7.7% in patients receiving conventional placebo (odds ratio 16.8, *p* < 0.001) [12]. An open label 24 week extension of this trial was completed and all study participants received burosumab. The authors noted increased fully-healed fractures and pseudofractures from baseline continued in subjects in the burosumab–burosumab arm (63% healed). Subjects enrolled in the placebo–burosumab arm experienced fracture healing that was comparable to those who initially started on burosumab at 24 weeks (35% healed) [17].

Pediatric clinical trials also demonstrated improvement in both laboratory and radiographic parameters [12,13,14]. Carpenter et al. compared the efficacy of a 2-week and 4-week dosing schedule in children aged 5–12 years using a 1 mg/kg dose [18]. Both dosing schedules led to significant improvement in Pi, 1,25-vitD, TRSS, and RGI-C; they also demonstrated an increase in walking distance utilizing the 6-min walk test (2-week 12% increase vs. 4-week: 10%) and an increased standing height physical ability (0.19 increase in z-score vs. 0.12). The authors did note that the 2-week dosing schedule provided a more sustained response with regards to maintaining Pi. Pi levels remained within or near normal limits at the end of the dosing interval, whereas children on the 4-week dosing schedule fell below the LLN. Conversely, 1,25-vitD responses were similarly maintained regardless of the dosing schedule utilized [14]. Another trial was completed in children aged 1–4 yrs using a dose of 0.8 mg/kg administered SC every 2 weeks. The dose was increased to 1.2 mg/kg if two consecutive Pi trough concentrations were below the LLN. Similar to previous studies, significant improvement was noted in both laboratory markers as well as radiographic findings [13].

An open-label, phase 3 pediatric clinical trial was conducted in patients aged 1–12 yrs. Subjects were randomized to receive either burosumab 0.8 mg/kg SC every 2 weeks or conventional therapy consisting of oral phosphate and active vitamin D replacement supplementation [10]. Dosage adjustments of burosumab were identical to those previously described above [10,11,12,13]. Oral phosphate and active vitamin D were also adjusted based on published recommendations [5,15,17]. Similar to previous studies, burosumab significantly improved both laboratory and radiographic markers of disease. Furthermore, improvements were noted in length and height Z scores (mean change 0.17 vs. 0.02, *p* = 0.049) and 6-min walking distance (mean change 9% vs. 2%, *p* = 0.0496). This was the first trial demonstrating superiority of burosumab compared with conventional therapy [13].

## 4. Pharmacology of Burosumab

Burosumab is available in three strengths: 10, 20 and 30 mg/mL, with an average wholesale price of $408 per milligram. Utilizing the recommended dose of 1 mg/kg for an adult patient weighing 70 kg, the anticipated average wholesale cost per dose is $28,560 [19]. While costly, burosumab is the only drug currently available on the market that is FDA approved to treat XLH [11]. Prior to the approval of burosumab, management options for XLH focused on treatment of electrolyte abnormalities with oral phosphate replacement and 1,25-vitD supplementation, whereas burosumab is able to target and block the pathway that is overexpressed in patients with XLH directly treating the underlying condition [20]. Based on the results reported in the phase 3 trial by Imel and colleagues, burosumab is superior to conventional therapy [10].

### 4.1. Dosage Recommendations

Burosumab is delivered via subcutaneous (SC) injection by a licensed healthcare provider. Prior to initiation of therapy, Pi levels should be drawn and evaluated, ensuring the level is lower than normal range based on the patient’s age [11]. Dosage recommendations for patients less than 1 year are not known as these patients were excluded from the clinical trials [10]. For children and adolescents 1–18 years of age, the dosage recommendation is 0.8 mg/kg of body weight, rounded to the nearest 10 mg given every 2 weeks via SC injection [11]. Dose may be adjusted if Pi remains below the LLN up to a max dose of approximately 2 mg/kg. If Pi increases to 5 mg/dL or greater, the next dose should be held and Pi reassessed in 4 weeks. Once Pi falls below the LLN, burosumab may be re-initiated at a reduced dose. The manufacturer provides recommended dose adjustment tables within the package insert [11]. The recommended dose for adults is 1 mg/kg SC, rounded to the nearest 10 mg, every 4 weeks. For both pediatric and adult patients, the maximum dose is 90 mg and the maximum volume for each injection is 1.5 mL [11].

The goal of therapy is to maintain Pi near the LLN based on the patient’s age to prevent hyperphosphatemia [21]. Pi should be drawn every 4 weeks with corresponding dose adjustments based on those levels. In adults, the first measurement of Pi should begin 2 weeks after initiation or dose changes, and in pediatrics the first measurement should begin 4 weeks after initiation or dose changes. If doses are missed, resume burosumab as soon as possible at the prescribed dose [11]. Burosumab is contraindicated in patients with severe renal impairment or end stage liver disease as both are associated with metabolic mineral disturbances, although no clinical trials occurred in these patients [11].

### 4.2. Pharmacokinetics

Following SC administration, burosumab follows linear pharmacokinetic modeling and reaches peak concentrations after 8 to 11 days (Table 2). Given burosumab is a monoclonal antibody, it is unlikely to be excreted intact into the urine. Metabolism of burosumab is not well defined, but it is hypothesized that it follows a similar pathway as other peptides and is catabolized into smaller peptides. A phase I clinical trial examined the effects of burosumab administered intravenously (IV) and SC across a variety of doses ranging from 0.003–0.3 mg/kg for IV administration and 0.1–1 mg/kg for SC administration [18]. SC administration had essentially complete bioavailability, and it increased the half-life from 8–11 days for the 0.1–0.3 mg/kg IV dosing range to 13–19 days for the 0.1–1 mg/kg SC dosing range. The authors note the extended half-life and bioavailability “support the practicality of a once-monthly” dosing regimen administered SC [18].

### 4.3. Pharmacodynamics

Two phase 1 adult studies concluded that a single injection of burosumab is sufficient to significantly increase the tubular reabsorption rate of phosphate regardless of SC or IV administration [18,22]. There were no recorded increases of nephrocalcinosis, serum parathyroid hormone, or creatinine. Additionally, after administration of a single dose, hypercalciuria, hypercalcemia, and anti-burosumab antibody formation were not detected. Peak Pi occurred seven days post injection and was maintained across a four-week injection schedule [23]. Serum levels of burosumab and Pi are linearly correlated (R = 0.812) which allow dosing adjustments of burosumab to be made based on pre-dose Pi concentrations [23].

### 4.4. Drug Interactions and Adverse Effects

Burosumab and its interaction with other drugs is unknown, however, it is not recommended to take oral phosphate or vitamin D analogs during treatment due to the risk of hyperphosphatemia and subsequent nephrocalcinosis. However, individuals with low vitamin D at certain ages may use a precursor supplement such as cholecalciferol or ergocalciferol to assist in maintaining normal vitamin D levels.

Similar types and quantity of drug related adverse effects (AEs) were found between the IV and SC groups in the phase 1 trial, albeit both with small sample sizes, 12 and 17 respectively. Additionally, this study measured for nephrocalcinosis, hypercalcemia, serum immature parathyroid hormone, and creatinine in both IV and SC groups and found no increases [18]. The type, quantity, and severity of drug-related AEs between control and burosumab groups in phase 2 and 3 clinical trials were largely similar, with the exception of injection site irritation being almost twice as common in pediatric patients as adults when compared to control. Hypersensitivity was also reported at a higher rate in pediatric patients than in adults but neither age group differed significantly compared to control. Nearly all drug-related AEs were rated as mild to moderate in severity. No AEs led to withdrawal of adults or children in the reported phase 2 and 3 clinical studies (Table 1), however three adults withdrew in two of the phase 1/2 studies due to urticaria (two patients) and restless leg syndrome (RLS) in the Imel et al. extension [16,19,23].

Injection site reaction, hypersensitivity, hyperphosphatemia, ectopic mineralization, and RLS were predefined as AEs of interest across the phase 2 and 3 clinical trials. While injection site reactions and hypersensitivity were common in pediatric trials (31–57.7% and 31–38%, respectively), there were no incidences of hyperphosphatemia or RLS [10,13,14]. As noted previously, incidence of injection site reactions and hypersensitivity were less frequent in the adult population (11.8% and 5.9%, respectively); however, the incidence of hyperphosphatemia and RLS was greater than that seen in the pediatric studies (5.9% and 11.8%, respectively) [12]. Ectopic mineralization was not noted in any of the phase 2 or 3 clinical trials, regardless of age [10,12,13,14].

## 5. Conclusions

XLH is a chronic condition primarily characterized by elevated FGF23 levels, causing hypophosphatemia and inappropriately low or normal levels of 1,25-vitD. Burosumab is a human monoclonal antibody against FGF23 and the first therapy targeted to the specific management of XLH. Its novel mechanism of action directly combats the hypophosphatemia in both pediatric and adult patients. The adverse effects of burosumab were rated mild to moderately severe, however, the occurrence of hyperphosphatemia was low. While burosumab therapy is costly, it is the only drug on the market to directly mitigate XLH’s clinical manifestations. Pending the results of ongoing clinical trials, future uses of burosumab may include other diseases that manifest due to elevated levels of intact FGF23 [24]. 

## 6. Data Sources and Study Selection

An English-language search of PubMed, MEDLINE, and clinicaltrials.gov was performed using the terms *burosumab*, *KRN23*, *X-linked hypophosphatemia*, and *FGF23* from January 2013 to February 2020 in March 2020. Articles including clinical trials of burosumab for the treatment of XLH in both adult and pediatric populations and review articles were evaluated for inclusion. References of articles were reviewed for additional data sources. Additional information was gathered from the manufacturer’s product labeling.

**Table 1:** Summary of clinical trials for burosumab.

**Cheong, 2019 (NCT02181764) [22]**	
**Design**	Phase 1, O, SC, single dose, dose escalation
**Population**	Adult N = 18; 0.3 mg/kg N = 6; 0.6 mg/kg N = 5; 1.0 mg/kg N = 7
**Efficacy Results**	Mean increase in serum Pi ^§^ 0.3, 0.6, 1.0 mg/kg (all increased) Mean TmP/GFR ^§^ 0.3, 0.6, 1.0 mg/kg (all increased) Mean serum 1,25(OH)2D ^§^ 0.3, 0.6, 1.0 mg/kg (all increased)
**Safety Results**	All AEs: 14 (78%) 0.3 mg/kg: 4 (67%) 0.6 mg/kg: 4 (80%) 1.0 mg/kg: 6 (86%) AEs related to burosumab: 5 (28%) 0.3 mg/kg: 3 (50%) 0.6 mg/kg: 0 1.0 mg/kg: 2 (29%) AE leading to discontinuation or death: 0 Most common AEs (≥10%): nasopharyngitis, upper respiratory tract infection, contusion, headache, back pain Most common drug-related AEs (≥10%): 0
**Carpenter, 2014 (NTC00830674) [18]**	
**Design**	Phase 1, R, DB, P, SC or IV, single dose
**Population**	Adult N = 38 IV N = 22 Burosumab, N = 17 Placebo, N = 5 SC, N = 16 Burosumab, N = 12 Placebo, N = 4
**Efficacy Results**	Serum Pi ^§^ IV: 0.003 ** 0.01 * 0.03 * 0.1 *** 0.3 mg/dL ** SC: 0.1, 0.3 *** 0.6 *** 1.0 mg/kg *** Serum 1,25(OH)_2_D ^§^ IV: 0.003, 0.01 * 0.03 *** 0.1 *** 0.3 mg/dL *** SC: 0.1, 0.3 *** 0.6 ** 1.0 mg/kg *** Serum calcium ^§^ IV: 0.003, 0.01 * 0.03, 0.1 * 0.3 mg/dL SC: 0.1, 0.3, 0.6, 1.0 mg/kg 24-h urine calcium ^§^ IV: 0.003, 0.01 ** 0.03 ** 0.1 *** 0.3 mg/dL ** SC: 0.1, 0.3, 0.6, 1.0 mg/kg 2-h urine calcium/creatinine ^§^ IV: 0.003, 0.01, 0.03, 0.1 * 0.3 mg/dL SC: 0.1 *** 0.3, 0.6 * 1.0 mg/kg
**Safety Results**	All AEs: IV Burosumab: 14 (82%) Placebo: 2 (40%) SC Burosumab: 10 (83%) Placebo: 2 (50%) AEs related to burosumab: 6 (21%) IV: 4 (24%) SC: 2 (17%) AE leading to discontinuation or death: 0 Most common AEs, IV (>10%): nausea and headache Most common AEs, SC (>10%): elevated serum amylase and back pain Most common drug-related AEs (>10%): nausea Most common drug-related AEs (>10%): elevated serum amylase
**Imel, 2015 (NCT01340482 and NCT01571596) [19]**	
**Design**	Phase 1/2, O, SC, 16 wks, dose escalation with sequential 12-month extension
**Population**	Adult, N = 28
**Efficacy Results**	Serum Pi ^§^** TmP/GFR ^§^** Serum 1,25(OH)_2_D ^§^** Serum procollagen type 1 ^§^** Osteocalcin ^§^** BALP ^§^**
**Safety Results**	All AEs: 27 (96%) AEs related to burosumab: 18 (64%) AE leading to discontinuation or death: 2 Moderate urticaria, severe restless leg syndrome Most common AEs (≥20%): arthralgia, nasopharyngitis, back pain, extremity pain, diarrhea, sinusitis, upper respiratory tract infection, dizziness and headache Most common drug-related AEs (≥20%): 0
**Zhang, 2016 (NCT01340482) [23]**	
**Design**	Phase 1/2, O, SA, SC, 16 wks, dose escalation
**Population**	Adult, N = 28
**Efficacy Results**	Serum Pi ^§^*** TmP/GFR ^§^*** Serum 1,25(OH)_2_D ^§^*** BALP ^§^*** P1NP ^§^*** Osteocalcin***
**Safety Results**	All AEs: Not reported AEs related to burosumab: Not reported AE leading to discontinuation or death: 1 Urticaria
**Ruppe, 2016 (NCT01340482) [16]**	
**Design**	Phase 1/2, O, SA, SC, 16 wks, dose escalation
**Population**	Adults, N = 28
**Efficacy Results**	SF36v2 mean: 1.77 * WOMAC mean: −3.8 *
**Safety Results**	All AEs: Not reported AEs related to Burosumab: Not reported AE leading to discontinuation or death: 1 Urticaria
**Portale, 2019 (NCT02526160) [17]**	
**Design**	Phase 3, R, DB, P, SC, 24 wks with O, 24 wk extension
**Population**	Adult, N = 134 Burosumab, N = 68 Placebo, N = 66
**Efficacy Results**	% achieved mean Pi above LLN DB Burosumab: 94.1% **** Placebo: 7.6% Extension Burosumab for 48 weeks: 84% Burosumab last 24 weeks: 89% Odds ratio of fracture or pseudofracture fully healing DB Burosumab: Placebo: 16.8 **** WOMAC: physical function & stiffness DB Burosumab: −3.11 * & −7.85 * Placebo: +1.79 & + 0.46 Extension Burosumab for 48 weeks: −7.76 *** & −16.03 **** Burosumab last 24 weeks: −8.18 **** & −15.82 **** Worst pain (BPI) DB Burosumab: −0.79 NS Placebo: −0.32 Extension Burosumab for 48 weeks: −1.09 **** Burosumab last 24 weeks: −1.18 ****
**Safety Results**	All AEs DB: 125 (93%) Burosumab: 64 (94%) Placebo: 61 (92%) Extension: 131 (97%) Burosumab for 48 weeks: 68 (100%) Burosumab last 24 weeks: 63 (95%) AEs related to burosumab DB Burosumab: 30 (44%) Placebo: 26 (39%) Extension Burosumab for 48 weeks: 42 (62%) Burosumab last 24 weeks: 32 (48%) AE leading to discontinuation or death: 0 Most common AEs (20%) DB Burosumab: 0 Extension Burosumab for 48 wks: nasopharyngitis, headache, arthralgia, tooth abscess Burosumab for last 24 wks: arthralgia Most common drug-related AEs: Not reported
**Insogna, 2018 (NCT02526160) [12]**	
**Design**	Phase 3, O, SA, SC, 48 wks
**Population**	Adult, N = 14
**Efficacy Results**	Mean % change osteoid volume/bone volume: −54% **** Bone turnover Osteoid thickness: −32% **** Osteoid surface/bone surface: −26% *** Median mineralization lag time: −83% Fracture or pseudofracture healing P1NP: +77% **** CTx: +36% ****
**Safety Results**	All AEs: 14 (100%) AE related to Burosumab: 10 (71%) AE leading to discontinuation or death: 0 Most common AEs: Not reported Most common drug-related AEs (≥10%): urticaria, abdominal pain, asthenia, injection site pain, injection site reaction
**Insogna, 2019 (NTC02526160) [15]**	
**Design**	Phase 3, R, DB, P, SC, 24 wks
**Population**	Adults, N = 134 Burosumab, N = 68 Placebo, N = 66
**Efficacy Results**	% achieved mean Pi above LLN Burosumab: 94.1% Placebo: 7.6% *** % maintaining PI above LLN before next dose Burosumab: 67.6% Placebo: 6.1% (significance not reported) Mean increase of 1,25(OH)_2_D ^#^ Burosumab: 25.5 pg/mL Placebo: 2.7 pg/mL *** Change in WOMAC stiffness Burosumab: −7.87 Placebo: +0.25 * % of fractures healed Burosumab: 43.1% Placebo: 7.7% ***
**Safety Results**	All AEs: Burosumab: 64 patients (94.1%) Placebo: 61 patients (92.4%) AE leading to discontinuation or death: 0 Most common AEs (≥10%): Back pain, nasopharyngitis, tooth abscess, injection site reaction, restless leg syndrome, headache, nausea, dizziness Most common drug-related AEs (≥10%): injection site reaction, restless leg syndrome
**Whyte, 2019 (NCT02750618) [13]**	
**Design**	Phase 2, O, SA, SC, 64 wks
**Population**	Children (1–4 yrs), N = 13
**Efficacy Results**	Mean increase of Pi: 0.89 mg/dL **** Mean increase of 1,25(OH)_2_D: 13 pg/mL **** Mean change of TRSS: −2.0 **** Mean change in RGI-C: +2.2 ****
**Safety Results**	All AEs: 13 (100%) AEs related to burosumab: 5 (38%) AEs leading to discontinuation or death: 0 Most common AEs (≥30%): Cough, pyrexia, upper respiratory tract infection, tooth abscess, rhinorrhea, vomiting, nasal congestion, diarrhea, pain in extremity, streptococcal pharyngitis, injection site reaction, hypersensitivity Most common drug-related AE (≥20%): injection site erythema and extremity pain
**Carpenter, 2018 (NCT02163577) [14]**	
**Design**	Phase 2, R, O, SC, 16 wk escalation with 48 wk treatment
**Population**	Children (5–12 yrs), N = 52 Every 2 wks, N = 26 Every 4 wks, N = 26
**Efficacy Results**	Mean change of TRSS from baseline ^†^ 2-wk: −1.06 *** 4-wk: −0.73 *** Mean change of RGI-C from baseline ^†^ 2-wk: +1.66 4-wk: +1.47 Mean increase of Pi All patients: 0.84 mg/dL Mean increase of 1,25(OH)_2_D All patients: 18 pg/mL
**Safety Results**	All AEs: 26 patients (100%) AEs leading to discontinuation or death: 0 Most common AEs, 2 wk dose schedule (≥30%): Injection-site reaction, headache, cough, nasopharyngitis, pain in extremity, upper respiratory tract infection, vomiting and pyrexia Most common AEs, 4 wk dose schedule (≥30%): Injection-site reaction, headache, cough, nasopharyngitis, pain in extremity, upper respiratory tract infection, vomiting, arthralgia, pyrexia, seasonal allergy Most common drug-related AEs: Not reported
**Imel, 2019 (NCT02915705) [10]**	
**Design**	Phase 3, R, O, AC, SC, 64 wks
**Population**	Children (1–12 yrs), N = 61 Burosumab, N = 29 Conventional, N = 32
**Efficacy Results**	Mean change of RGI-C ^†^ Burosumab: +1.9 Conventional: +0.8 **** Mean change of TRSS ^†^ Burosumab: −2.0 Conventional: −0.7 **** Mean increase in Pi Burosumab: 0.92 mg/dL Conventional: 0.20 mg/dL Mean increase of 1,25(OH)_2_D Burosumab: 30 pg/mL Conventional: 18 pg/mL
**Safety Results**	All AEs: Burosumab: 29 patients (100%) Conventional: 27 patients (84%) AE leading to discontinuation or death: 0 Most common AEs (≥30%): Pyrexia, injection site reaction, cough, arthralgia, vomiting, nasopharyngitis, hypersensitivity, pain in extremity, headache, injection site erythema, dental caries

*Abbreviations*: AC, active-controlled; AEs, adverse events; BALP, bone alkaline phosphatase; CTx, C-terminal telopeptide; DB, double-blind; IV, intravenous; LLN, lower-limit of normal; NS, not significant; O, open-label; P, placebo-controlled; P1NP, N-terminal propeptide type 1 collagen; Pi, inorganic phosphate; R, randomized; RGI-C, Radiographic Global Impression of Change score; SA, single arm; SC, subcutaneous; SF36v2, self-perceived limitations due to physical health; TRSS, Thacher rickets severity score; WOMAC, Western Ontario and McMaster Universities Arthritis Index. ^#^ comparison reported at 22 weeks; ^†^ comparison reported at 40 weeks; ^§^ exact values not reported; * *p* < 0.05, ** *p* < 0.01, *** *p* < 0.001, **** *p* < 0.0001; note: *p*-values not reported for all comparisons by study authors.

**Table 2:** Pharmacokinetic properties for burosumab.

T_max_	8 to 11 days
Vd	8 L (based on a 70 kg adult)
Steady-State Trough	5.8 ± 3.4 mcg/mL (adult) 15.8 ± 9.4 mcg/mL (pediatrics 5–12 years old) 11.2 ± 4.6 mcg/mL (pediatrics 1–4 years old)
Metabolism	Not characterized. Likely broken down into small peptides and amino acids via catabolism
Half-Life	19 days
Clearance	0.290 L/day
Special Populations	Unknown whether renal or hepatic dysfunction affects pharmacokinetic properties Increased body weight results in increased clearance and Vd
Excretion into Breastmilk	No information is available at this time. Because it is a large peptide, it is likely only present in very small amounts and poorly orally absorbed due to degradation in the infant’s GI tract [25]
Drug interactions	No studies conducted to date, though unlikely to be affected by CYP inhibitors or inducers

*Abbreviations*: CYP, Cytochrome P450; GI, Gastrointestinal; T_max_, time to maximum concentration; Vd, Volume of Distribution.

## Data Availability

Not applicable.
